# The epidemic of sexually transmitted infections in China: implications for control and future perspectives

**DOI:** 10.1186/1741-7015-9-111

**Published:** 2011-10-06

**Authors:** Xiang-Sheng Chen, Rosanna W Peeling, Yue-Ping Yin, David C Mabey

**Affiliations:** 1 National Center for STD Control, Chinese Academy of Medical Sciences and Peking Union Medical College Institute of Dermatology, Nanjing, China; 2 Clinical Research Department, Faculty of Infectious and Tropical Diseases, London School of Hygiene & Tropical Medicine, London, UK

## Abstract

China has experienced an increasing epidemic of sexually transmitted infections (STIs), including HIV. High risk groups likely to be infected include female sex workers (FSWs) and their clients, men who have sex with men (MSM), drug users and migrant workers. Prevention can be achieved through education of the population, condom promotion, early detection of symptomatic and asymptomatic people, and effective diagnosis and treatment of these patients and their partners. This article aims to describe the profile of the epidemic in high-risk groups in China as well as to detail the contributing factors and the implications for control. Programmes for the control of STIs should be immediate priorities in China, and primary and secondary prevention strategies are vital to this process.

## Introduction

Sexually transmitted infections (STIs) have been recognized as major public-health problems in China since they re-emerged with the introduction of the open door policy and economic liberalization; they rapidly spread throughout the country from the early 1980s onwards [1,2]. Although syphilis had been eliminated from China in the 1960s by making free screening and treatment available to all [3], it is now among the top three reported notifiable diseases in the country. Female sex workers (FSWs) and their clients, men who have sex with men (MSM), drug users, migrant workers and youth are recognized high risk groups for STIs. There are an estimated 2.8 to 4.5 million FSWs and 21.9 to 37.4 million clients of FSWs [4], 2 to 10 million MSM [5], and 130 million migrant workers in China [6]. HIV/AIDS and other STIs have been spreading from high risk populations to the general population [7]. While potential determinants of the spread of HIV/AIDS, and responses to the HIV/AIDS epidemic have been outlined in other reviews [8,9], the purpose of this review is to provide an overview of the current epidemic of other STIs, such as syphilis, gonorrhea, chlamydia and herpes simplex virus 2 (HSV-2) infections, in mainland China from the perspective of disease burden, prevalence in high-risk groups, and the implications for control.

### National surveillance data

Accurate surveillance data of STIs are crucial for developing a prevention and control programme and providing valuable measures to assess the impact of behavioral interventions and measure the likelihood of sexual transmission of HIV. In China, HIV/AIDS, syphilis and gonorrhoea are reportable STIs according to the Law of the People's Republic of China on Prevention and Treatment of Infectious Diseases, and it is mandatory to report new cases to the national surveillance system. The number of cases with primary (characterized by the appearance of a painless ulcer, called a chancre, about three weeks after sexual contact with an infected person) and secondary syphilis (characterized by a rash involving the palms and soles, accompanied by fever, sore throat, hair loss and swelling of lymph nodes about four to six weeks after primary syphilis), and the reported incidence are used for monitoring the epidemic. The reported incidence of primary and secondary syphilis (reflecting the intensity of recent transmission) was 11.7 cases per 100, 000 residents in 2009, increasing by 2.1 times since 2005 [10]. However, the reported incidence of gonorrhoea has decreased by about 30% [10]. Such "scissors-like differential" phenomenon has also been found in other countries, such as the United States [11].

### Incidence studies of STIs among high risk subgroups

National surveillance data based on case-reports in clinical settings can give an indication of the incidence of various STIs within the population but they are critically subject to the symptomatic nature of the diseases, STI care-seeking behaviors of patients, and the quality of the care-reporting system. Prospective cohort studies can provide the best setting in which to estimate the true incidences of STIs. There are a few cohort studies among MSM and FSWs that observe incidence rates of STIs in China. However, all of these studies focus on syphilis as shown in Table 1[12-18]. The rates of MSM were different from area to area, ranging from 7.6 to 38.5/100 person-years (PYs). Sourcing male sex partners in parks, public washrooms, bathhouses, or gay saunas was significantly associated with increased risk of acquiring syphilis infection in MSM [12-14]. The incidence of syphilis among FSWs varied substantially, from 1.1/100 PYs in one study [17] to 6.2/100 PYs in another [18]. The risk of acquiring syphilis among FSWs was critically dependent on the venue where they solicited clients. A recent prospective cohort study conducted in three cities from June 2009 to March 2011 (Chen XS, unpublished data) showed that the high-tier FSWs (who solicited clients in venues such as karaoke bars or star hotels) had much lower incidence of syphilis (0.7/100 PYs) than the middle-tier FSWs (who solicited clients in venues such as hair salons, barber shops, massage parlors, foot bath shops, or roadside shops) or the low-tier FSWs (who solicited clients in outdoor venues) (6.8 to 7.4/100 PYs). The higher syphilis risk associated with low-tier FSWs may be related to greater numbers of clients per day [19], lower rates of condom use with clients that can pay extra for unsafe sex, or more frequently changing work locations [20,21]. More studies on the incidence of other STIs and parameters related to the risk of new infections of STIs in various high-risk groups are needed to well understand the epidemic in China.

**Table 1:** Incidence density of syphilis infection among MSM and FSWs - results from cohort studies *^a^*

Study	Study year	Study location	Sample size	Incidence density per 100 person- years (95% CI)*^b^*	Risk factors
Men who have sex with men					
Li SM, *et al*. [12]	2008	Beijing	525	9.3 (5.9 to 12.8)	Sourcing male sex partners in parks, public washrooms or bathhouses Performing rectal douching after homosexual anal intercourse
Hao C, *et al*. [14]	2011*^c^*	Nanjing	348	7.6 (2.6 to 12.5)	Sourcing male sex partners mostly from gay saunas High monthly income; Self-identification of homosexual orientation
Li D *et al*. [15]	2006 to 2007	Beijing	507	16.9 (12.4 to 21.3)	Sourcing male sex partners in bathhouses/public washrooms/parks Having less education Drinking alcohol four or more times monthly
Xu JJ *et al*. [16]	2006	Shenyang	218	38.5 (27.7 to 50.2)	Being married Having more than five male sexual partners within the past 12 months
Zhang M *et al*. [13]	2007	Shenyang	229	21.6 (14.4 to 30.4)	No data available
	2008		598	26.0 (21.9 to 30.9)	
	2009		455	29.8 (21.8 to 38.7)	
Female sex workers					
Wang HB *et al*. [17]	2006 to 2007	Kiayuan	492	1.1	Being elder Having less education Having more clients Inconsistent use of a condom
Tian LG *et al*. [18]^6^	2004 to 2005	Xichang	343	6.2	No data available.

*^a ^*FSWs, female sex workers; MSM, men who have sex with men

*^b ^*CI, confidence interval.

*^c ^*This is year the study was reported as the study year was not reported in the paper.

### Prevalence of sexually transmitted infections among subgroups

#### Syphilis

Syphilis is an STI caused by the spirochete bacteria *Treponema pallidum *subspecies *pallidum*. Sexual contact is the primary route of transmission but it may also be transmitted from mother to child during pregnancy, resulting in adverse pregnancy outcomes. Syphilis is one of the most common STIs in China. Although the substantial heterogeneity in design of the studies prevented a more rigorous meta-analysis, a systematic literature review of published work during 2000 to 2005 has provided comprehensive information on syphilis prevalence among different populations and suggested the following prevalence of syphilis infection in specified populations: antenatal women 0.4%; premarital individuals 0.7%; voluntary blood donors 0.4%; commercial blood donors 2.9%; possible FSWs 0.8%; incarcerated FSWs 12.5%; FSW clients 3.0%, drug users 6.8%; and MSM 14.6% [22]. During the years 2000 to 2005, syphilis rates had increased in all these groups, with the greatest increase in men who have sex with men (4.5% per year) followed by incarcerated FSWs (1.4% per year) and drug users (1.0% per year) [22].

FSWs and MSM are the subgroups at highest risk of syphilis infection, as shown in Table 2. A meta-analysis of published studies targeting MSM in 12 Chinese cities reported a summary prevalence of 9.1% (95% CI, 7.6 to 10.8%) in this group [23]. However, the heterogeneity of the included studies in terms of sampling methods, sampling size and study locations needs to be taken into account when explaining some of the inconsistencies observed between studies. Factors significantly associated with syphilis infection were older age, less education, having sought male sex partners through bathhouses/public washrooms/parks, and alcohol consumption in MSM [24]. A recent systematic review we did with 72 published studies reporting syphilis prevalence among FSWs in China found that the median prevalence was 6.3% with an interquartile range (IQR) of 3.7 to 11.8% in FSWs [25]. Some studies among FSWs from low-tier venues found extremely high syphilis prevalence, ranging from 10 to 38% [26]. In most of the studies in FSWs or MSM, syphilis was significantly associated with HIV infection [27,28].

**Table 2:** Prevalence of chlamydia, gonorrhoeae, and herpes simplex virus type 2 infections in different subgroups

Study	Study year	Study location	Population *^a^*	Sample size	Prevalence (%)*^b^*		
					
					CT	NG	HSV-2
Jin × *et al*. [29]	2008	Kaiyuan	Urban FSWs	568	17.4	8.3	
Wang H *et al*. [30]	2006	Kaiyuan	Urban FSWs	737	25.9	8.3	68.1
Chen XS *et al*. [31]	2006	Shenzhen	Urban FSWs	70	25.7		
Ngo TD *et al*. [49]	2008*^c^*	Kunming	Urban FSWs	500			33.0
van den Hoek A *et al*. [32]	1998 to 1999	Guangzhou	Urban FSW	966	32.2	8.8	
Xu JJ *et al*. [33]	2006	Gejiu	Rural FSWs	96	46.3	36.8	70.8
Chen XS *et al*. [27]	1999 to 2000	Kunming	FREC FSWs	505	58.6	37.8	65.1
Chen XS *et al*. [31]	2006	Shenzhen	FREC FSWs	547	10.8		
Jiang J *et al*. [34]	2003	Multiple cities	MSM	112	8.0	2.7	7.8
Feng Y *et al*. [28]	2007	Chengdu	MSM	513			28.1
Li JH *et al*. [35]	2008 to 2009	Shenzhen	MSM (rectal site)	131	24.4	0	
	2008-2009	Shenzhen	MSM (urethral site)	131	5.3	1.5	
He N *et al*. [36,50]	2005*^c^*, 2009*^c^*	Shanghai	Male rural migrants	1, 086	1.0	0.5	5.5
Wang W *et al*. [37]	1999 to 2000	Multiple cities	Male migrants	234	1.8	0	
			Male urban non-migrants	1, 095	2.2	0	
			Male rural non-migrants	359	2.1	0	
			Female migrants	198	4.8	0.2	
			Female urban non-migrants	1184	5.1	0.3	
			Female rural non-migrants	355	1.6	0	
Wu Z *et al*. [38]	2007*^c^*	Fuzhou	Male market vendors	2, 132	6.3	0.6	4.7
			Female market vendors	2, 378	11.2	1.4	8.2
Detels R *et al*. [39]	2003*^c^*	Eastern China	Male market vendors	617	5.4	0.8	6.4
			Female market vendors	699	13.1	1.2	12.0
Chen XS *et al*. [40]	1999 to 2000	Tongling	Truck drivers	550	10.6	8.1	4.4
Zhang GL *et al*. [41]	2006	Gejie	Male miners	1, 796	4.8	0.8	9.6
Xu JJ *et al*. [33]	2006	Gejiu	Male miners	339	6.5	2.1	14.9
Chen XS *et al*. [42,51]	2002	Fuzhou	Urban pregnant women	504	10.1	0.8	10.8
Shi XB *et al*. [43]	2001*^c^*	Changsha	Urban pregnant women	72	13.9		
Li JM *et al*. [52]	2004 to 2006	Southern China	Pregnant women	1, 740			23.6
Parish WL *et al*. [44]	1999 to 2000	Multiple cities	Community men	1, 688	2.6	0.02	
	1999 to 2000	Multiple cities	Community women	1, 738	2.1	0.08	

*^a ^*FSWs, female sex workers; MSM, men who have sex with men; FREC, female sex workers at re-education center.

*^b ^*CT, *Chlamydia trachomatis *infection detected by nucleic acid amplification test (NAAT); NG, *Neisseria gonorrhoeae *infection detected by NAAT; HSV-2, herpes simplex virus type 2 infection detected by enzyme Immunoassay (EIA).

*^c ^*This is the study was reported as the study year was not reported in the paper.

#### Chlamydia

Chlamydia is a common STI caused by the bacterium *Chlamydia trachomatis *(CT). It is the most prevalent bacterial STI in China, but it has not yet been included as a reportable STI in the national STI surveillance program in China. Therefore, available data on the prevalence of CT infection are limited to a few *ad hoc *prevalence surveys among different populations, some of which used highly sensitive nucleic acid amplification tests (NAAT) to diagnose the infection (Table 2) [27,29-44]. A large population-based study in 1999 to 2000 among 3, 426 Chinese residents found a prevalence of 2.1% among men and 2.6% among women [44]. Of these, 10% of men and 4% of women reported having sex with two or more noncommercial sexual partners in the last year. Risk of infection among men younger than 45 years was significantly associated with unprotected sex with FSWs, while the risk for women was significantly associated with the behavior and income of their spouses or steady partners.

FSWs have the highest prevalence of CT infection, particularly in those recruited from the female re-education centers but, interestingly, a baseline study of our Mega project among FSWs from different typologies of venues did not find any significant difference in CT infection between high-tier (17.0%), middle-tier (16.5%) and low-tier FSWs (18.7%), (unpublished data, Chen XS).

Regarding the MSM population, a study of simultaneously collected urethral and anorectal specimens among participants in Shenzhen indicated a much higher prevalence of anorectal CT infection (24.4%) than urethral infection (5.3%) [35]. Lymphogranuloma venereum (LGV) proctitis is caused by CT genotype L and is endemic among MSM in western society, but it has not been reported to be found among MSM in China, although there have been sporadic cases reported in STD clinics [45].

Migrant workers have a modest prevalence of CT infection (5 to 10%), with a higher prevalence in females [37-39]. Most strikingly, pregnant women have a relatively high prevalence of CT infection, ranging from 10 to 13% in two studies [42,43]. Untreated CT infection can lead to pelvic inflammatory disease (PID) in women and premature delivery in pregnancy. Babies who are born to infected mothers can get CT infections in their eyes and respiratory tracts. Screening for CT infection at the first prenatal visit has been recommended in the United States [46], but this recommendation was recently re-evaluated in the UK [47].

#### Gonorrhoea

Gonorrhea is a common STI caused by the bacterium *Neisseria gonorrhoeae *(NG). NG infection is prevalent in high risk groups, particularly among FSWs (8 to 40%) (Table 2) [27,29,30,32,33], although the incidence based on the case reports in clinics has shown a declining trend over the past decade [10]. In other high risk groups such as MSM and migrant workers, the prevalence was reported to be less than 5% [34-39]. The overall prevalence of NG infection in the general population was 0.08% for women and 0.02% for men [44]. One of the major concerns about the NG epidemic is the emergence of drug resistance. Out of 1, 398 gonococcal isolates obtained from STD clinics in 15 cities in China in 2009, 37.4% were penicillinase-producing NG (PPNG), 48.5% were plasmid mediated tetracycline-resistant NG (TRNG), and 96.6% had chromosomal resistance to ciprofloxacin (QRNG). Fourteen ceftriaxone-resistant NG strains (1.0%) and five spectinomycin-resistant NG strains (0.4%) were sporadically detected in some areas, while increasing numbers of NG strains with decreased susceptibilities to ceftriaxone, which is widely used for the treatment of gonorrhoea, have been isolated in most of the surveillance sites [10].

#### Herpes simplex virus type 2 infection

Herpes simplex virus type 2 (HSV-2) primarily infects the genital mucosa and is the main cause of genital herpes. HSV-2-seropositive individuals have a lifelong risk of infecting their sexual partners [48]. Like other STIs, HSV-2 infection was most prevalent among FSWs, with a seroprevalence of 30 to 70% (Table 2) [27,30,33,49]. A modest seroprevalence of HSV-2 infection (8 to 30%) occurred among MSM in previous studies [28,34]. However, a relatively high seroprevalence in a few studies was found in pregnant women (11 to 24%) [51,52], as compared with that in the migrant population (less than 12%) with a similar age distribution [38,39].

In summary, it is known from the available data that: (1) the MSM population has the highest syphilis prevalence followed by FSWs, and the MSM recruited from bathhouses, public washrooms or parks and the low-tier FSWs who solicited clients on streets or other outdoor settings have a higher prevalence than their counterparts from other venues; (2) the FSW population has the highest prevalence of chlamydial infection, and the prevalence of rectal chlamydial infection among MSM populations is higher than that of urethral infection; (3) gonococcal infection is relatively low in high-risk groups but increasing emergence of drug resistance is a major concern; and (4) seroprevalence of HSV-2 infection is high not only in high-risk groups but in the general population.

### Implications for control

Evidence from the data of the epidemiological trends in reported syphilis cases, and the prevalence and incidence estimates of STIs among different populations suggests that STIs are a major public health concern in China. Strong evidence has supported biological mechanisms through which STIs, particularly ulcerative STIs, can facilitate HIV transmission by increasing both HIV infectiousness and HIV susceptibility [48,53]. A rapid increase of the reported STI cases, a high prevalence of STIs, and a large number of the population at high risk will further fuel the sexual transmission of HIV in China. Lessons learned from some countries where sex work is legal suggests that sex workers who engage in commercial sex but do not participate in the licensing regime are usually less healthy than licensed sex workers because they are usually excluded from reputable health services, and easily missed by health promotion programmes [54]. Sex work or prostitution is illegal in China, although there is still heated debate among scholars on the current crackdown policy on the sex industry [55].

#### Primary prevention

Health education messages and structural interventions to promote safe sex and increase access to condoms can help to prevent all STIs. In China, with its huge population of migrants from rural communities to more affluent urban centres, health education will need to begin in school. It will be more difficult to target high risk groups, such as low-tier FSWs, once they are established in their trade. Although the national behavioral data have suggested an increasing rate of condom use among FSWs [7], the rate among FSWs working in the middle- or low-tier venues remains quite low [56], whereas sex partner change rate remains high [19]. The internet could be used for health promotion among MSM, who often use it to find partners. Media campaigns, using celebrities on radio, television or mobile phone technology, have been effective in promoting condom use in China and could be used to deliver sexual health messages.

#### Secondary prevention

Early detection and treatment of individuals with STIs is an essential part of an STI control strategy as it interrupts the chain of transmission. This can be facilitated through clinical and partner notification services for those with symptoms of STI, and through screening programmes for asymptomatic individuals at high risk. Clinical services must be accessible to and affordable by those at high risk, who are often poor migrants. STD clinics are an important access point for people at high risk of both STIs and HIV. Identifying people with STIs allows for not only the benefit of treating the STIs, but for counseling and education, condom promotion, HIV testing, and partner notification for STIs or HIV infection. Since many doctors working in STI clinics do not routinely provide these services, there is a need for continuing medical education to encourage them to do so. Since many patients in China seek treatment for STIs in the private sector, it will be important to include doctors and pharmacists working in the private sector in these educational programmes [57].

Responding to the importance of providers in expanding HIV and syphilis testing, provider-initiated testing and counseling (PITC) focused on promoting HIV and syphilis testing by physicians has been introduced in China after pilot studies. The implementation of PITC is critically important in public STI clinics, which see a large number of patients at risk for sexually transmitted HIV infection. The STI prevention and control program needs to be strengthened and integrated with HIV control, and must be accessible to all. Key elements of the program should include health-promotion, improved coverage of screening, improved access to treatment and partner notification, community mobilization and stigma reduction, improved surveillance, and monitoring and evaluation.

Introduction of rapid point-of-care (POC) tests into clinic and outreach services targeting high risk groups will provide a new and unique opportunity for ensuring STI detection and treatment. Recent evidence from a pilot study supported by the WHO in two cities in China indicated that some 75% of FSWs accepted to be tested with rapid syphilis tests (RST) in sex work settings, and about 50% of those with positive RST were successfully referred for final diagnosis and treatment [58]. The availability of quality-assured rapid POC tests in public and private clinics, and the integration of POC screening into outreach services targeting high-risk groups, will help promote coverage and accessibility of early detection and timely treatment of STIs.

#### Changes in policy

Without efforts to destigmatize STI and HIV and to create good linkages between outreach services and clinical care, it is unlikely that control of STIs among high-risk groups will easily be achieved. Many of the FSWs who refused POC testing in our pilot study did so because they feared incarceration. Recently, the State Council of China issued a notice on further strengthening HIV/AIDS responses [59], in which HIV and syphilis are both considered as important public issues that should be given equal attention in providing testing and counseling services, preventing mother-to-child transmission, and conducting comprehensive interventions. Specifically, China's recently launched 10-year national syphilis control and prevention plan includes the national milestone of achieving an explicit decline in the reported syphilis incidence and the elimination of congenital syphilis by 2020, indicating specific targets for percentages of target populations educated about syphilis, tested and treated for syphilis [60].

#### Improved surveillance

In China, some subgroups linked to high risk of STIs, such as low-tier FSWs, MSM and migrant workers, have been less well represented in the surveillance program, and the surveillance of other important STIs, such as CT, NG and HSV-2 infections, has not been sufficiently integrated. These issues should be critically considered to improve the current STI and HIV surveillance programmes.

### Future perspectives

Syphilis had been eliminated from China in the 1960s by making free screening and treatment available to all [2,3]. Although much has changed in the past 60 years, universal access to reliable diagnosis and effective treatment would have a major impact on the current STI epidemic in China. Although the Chinese government has demonstrated its commitment and willingness to take action to control syphilis and other STIs, including development and promulgation of national policies and programmes on STI control and integration of STI control into HIV prevention programmes [59,60], there still remain several major challenges to effective control of STIs in China. Stigma and discrimination continue to hinder the uptake of testing services, medical care, partner notification, and behavioral interventions. Limitations in prevention and treatment services within the healthcare system also exacerbate these problems. As a result, many people infected with STIs cannot be diagnosed and treated in a timely manner to protect themselves and their partners. Multi-sectoral responses and support from relevant agencies are not sufficient for translating the plan into action. Further implementation research is urgently needed to address the questions, for example, on the possibility of promoting structural interventions targeting different typologies of FSWs or MSM, in which STI and HIV awareness and prevention can be integrated, the development of innovative strategies to reach marginalized populations in terms of conducting prevention outreach, the applicability to Chinese social and cultural contexts of condom use and other prevention approaches, and the mobilization of local civil society organizations and social network groups for prevention efforts. In addition to strengthening the current surveillance programme, more observation studies on STI disease burden, risk factors and interventions are needed to provide a solid base for planning and policy change. Evaluation research, including cost-effectiveness and modeling studies on prevention strategies among target groups and in different scenarios, is urgently needed in order to know what works and what is worth taking to scale. Policy research, including the recognition of the current gaps in policy, the mechanism of translating evidence to policy and ensuring the policies are implemented is also required.

## Conclusions

In conclusion, the epidemiologic data presented in this paper show the presence of substantial STI epidemics in China, particularly among high-risk groups, and the underlying potential for continued growth in the future. However, further epidemiological studies and implementation research are urgently needed for better understanding of the epidemic and its risk factors, development of the prevention programmes and formulation of control policies. Expanding the comprehensive control programme consisting of primary and secondary prevention strategies will help ensure that STI transmission can be prevented and the infected individuals can be diagnosed in a timely fashion and treated. Cost-effectiveness studies and further policy work will help justify the expanded resources necessary to implement the programme. Integration of STI control into HIV/AIDS prevention and control programme represents a feasible and scalable intervention to thwart China's STIs epidemic.

## Abbreviations

CT: *Chlamydia trachomatis*; FSWs: female sex workers; HSV: herpes simplex virus; LGV: lymphogranuloma venereum; MSM: men who have sex with men; NAAT: nucleic acid amplification test; NG: *Neisseria gonorrhoeae; *PID: pelvic inflammatory disease; PITC: provider-initiated testing and counseling; POC: point-of-care; PPNG: penicillinase-producing NG; PY: person-year; QRNG: chromosomal resistance to ciprofloxacin; RST: rapid syphilis test; STI: sexually transmitted infection; TRNG: tetracycline-resistant NG.

## Competing interests

The authors declare that they have no competing interests.

## Authors' contributions

XSC prepared the first draft of the manuscript and revised the manuscript according to reviewers' comments, and YPY helped to collect some epidemiological data. All authors made comments on the manuscripts and approved the final version.

## Pre-publication history

The pre-publication history for this paper can be accessed here:

http://www.biomedcentral.com/1741-7015/9/111/prepub
